# Systematic review and meta-synthesis of coping with retinitis pigmentosa: implications for improving quality of life

**DOI:** 10.1186/s12886-019-1169-z

**Published:** 2019-08-13

**Authors:** Gulcan Garip, Atiya Kamal

**Affiliations:** 1 University of Derby Online Learning, University of Derby, Kedleston Road, Derby, DE22 1GB England; 2 Department of Psychology, Birmingham City University, The Curzon Building, Birmingham, B4 7BD England

## Abstract

**Background:**

Retinitis pigmentosa (RP) are a group of incurable and inherited eye conditions, and the leading cause of inherited blindness in people under the age of 60. The aim of this systematic review and meta-synthesis was to present a comprehensive overview of qualitative papers on experiences and coping strategies of adults living with RP, and how these influence quality of life.

**Methods:**

A pre-registered search strategy was applied in nine databases and 12 articles met eligibility criteria. Studies included were from Australia, Brazil, Ireland, Netherlands, Republic of Korea, United Kingdom, and USA. The overall sample was based on 126 people with RP (ages ranging from 18 to 85; at least 65 female). Principles of meta-ethnography were used to synthesise the articles revealing five higher-level meta-themes.

**Results:**

The five higher-level meta-themes were, 1) managing identity: making sense of RP, managing autonomy and independence; 2) living with RP: practical and emotional issues; 3) experiences with healthcare professionals and other social support; 4) adaptive and maladaptive coping strategies; and 5) impact of RP on work and career. A conceptual model was developed by grouping higher-level meta-themes as intra- and inter-individual factors and how they may be implicated with coping strategies and quality of life.

**Conclusions:**

This review established factors that can be explored as potential psychosocial influences in the relationship between coping strategies and quality of life in people with RP. Further understanding of these factors and mechanisms can help inform intervention development to support adaptive coping in living with RP and positively impact quality of life.

## Background

Retinitis pigmentosa (RP) is a group of incurable and inherited eye conditions, affecting 1 in 4000–5000 people, and is the leading cause of inherited blindness in people under the age of 60 [1]. Visual impairment is caused by progressive degeneration of retinal photoreceptors resulting in permanent changes to vision [1]. Age of onset varies from early childhood to middle ages, with most people becoming legally blind by their 40s or 50s. People with RP become more dependent on others for daily tasks [2], are less likely to contribute to the workforce and society, and are more likely to use health services [3], compared to those without the condition [4]. Adults living with RP are more likely to experience higher levels of distress and depression [5], and report lower levels of quality of life compared to adults living without the condition or with other health conditions (e.g. diabetes [6]). Supporting people with RP to develop approaches to cope, manage, and adapt to living with RP could improve quality of life in individuals living with the condition, as well as ameliorate the costs to society.

Physical symptoms of RP initially include night blindness or loss of visual acuity, gradually progressing to loss of peripheral vision, difficulty seeing in poor light, and for some, may result in blindness; the progression of the condition may happen gradually over a number of years or a relatively more rapid loss of vision in other cases [1]. The psychological impact of vision loss on wellbeing in individuals across ten European countries was associated with poorer emotional health and all aspects of wellbeing, and the authors conclude there is much potential for improving both eyesight and quality of life in people with impaired vision [3]. RP causes greater emotional and psychological impact compared to people with major blinding conditions such as diabetic retinopathy and age-related macular degeneration, as these conditions are mostly treatable and occur later in life, whereas RP has a relatively earlier onset and is incurable [7*]. The earlier onset of RP can have a negative impact on career trajectories and result in greater financial burden at an individual and societal level [8].

Coping strategies are a key factor in adapting to stressors, such as progressive vision loss. Most research on coping has relied on the Stress and Coping Model [9], which distinguishes between problem-focused (e.g. changing behaviours to deal with the problem), emotion-focused (e.g. regulating emotions in response to the problem), and avoidance coping (e.g. ignoring or distracting self from the problem) strategies. Problem-focused and emotion-focused coping strategies may be adaptive under different conditions and it is useful to understand any maladaptive coping strategies used by people with RP to be able to address these issues. This paper sets out to give an overview of the coping strategies used by people with RP, with the intention of informing the development of appropriate interventions for individuals with RP.

Various visual impairments (e.g. diabetic retinopathy, age-related macular degeneration, etc.) have different symptoms and progression trajectories; in fact, there is also much variation among progression and symptoms experienced among people with RP [1]. Qualitative studies of people’s experiences of living with different visual impairments exist and may be problematic for distinguishing condition specific issues. To minimise variability among different visual conditions, we have opted to focus on the experiences of people with RP to develop condition specific recommendations for improving quality of life in this group.

In this paper, we adopt the health-related quality of life definition proposed by Leidy and colleagues [10], which posits quality of life as a person’s subjective perception of the impact of health status, including disease and treatment (in this case RP), on physical, psychological, and social functioning and well-being. We opted for a more global and multifaceted definition than vision-related quality of life, which specifically focuses on the extent to which vision impacts life satisfaction and the ability to complete daily activities, as this may miss quality of life issues indirectly linked with vision impairment due to RP [11].

Understanding the experiences and coping strategies used among adults with RP could help inform the development of interventions and guide suggestions for healthcare providers to improve quality of life in this population. Bittner and colleagues [12*] state that improving health and quality of life in patients with chronic, disabling conditions, such as RP, involves more than identifying treatments and cures. Therefore, there is a need for a holistic and multidisciplinary approach to understand and provide support for people with RP, that does not only focus on the medical and physiological aspects related to the condition, but also takes into consideration, psychosocial and organizational factors as well [13, 14].

The aims of this systematic review and meta-synthesis were to bring together qualitative findings on coping strategies and quality of life in people with RP. The objectives were:

- to identify coping strategies used by adults living with RP;
- and to present how these findings may inform interventions to improve quality of life in this population.

## Methods

This systematic review was conducted within the Preferred Reporting Items for Systematic Reviews and Meta-Analyses standards (PRISMA [15],) and followed a predetermined registered protocol (PROSPERO: CRD42018098585) (Garip G, Kamal A: A systematic review and meta-synthesis of coping with retinitis pigmentosa: implications for improving quality of life, Unpublished). The review was conducted in four stages: (1) a systematic search was carried out to identify potential eligible papers, (2) initial records retrieved were screened for relevance based on title and abstract and resulting articles were fully read to determine eligibility for the purposes of the review, (3) papers were independently quality appraised by the authors according to the Critical Appraisal Skills Programme (CASP) criteria [16], 4) findings from eligible papers were extracted and synthesised according to principles of meta-ethnography, which is an interpretivist approach to derive understanding from different studies on a topic of interest, in this case, coping strategies of people with RP [17, 18].

### Search strategy and screening process

**Fig. 1:**
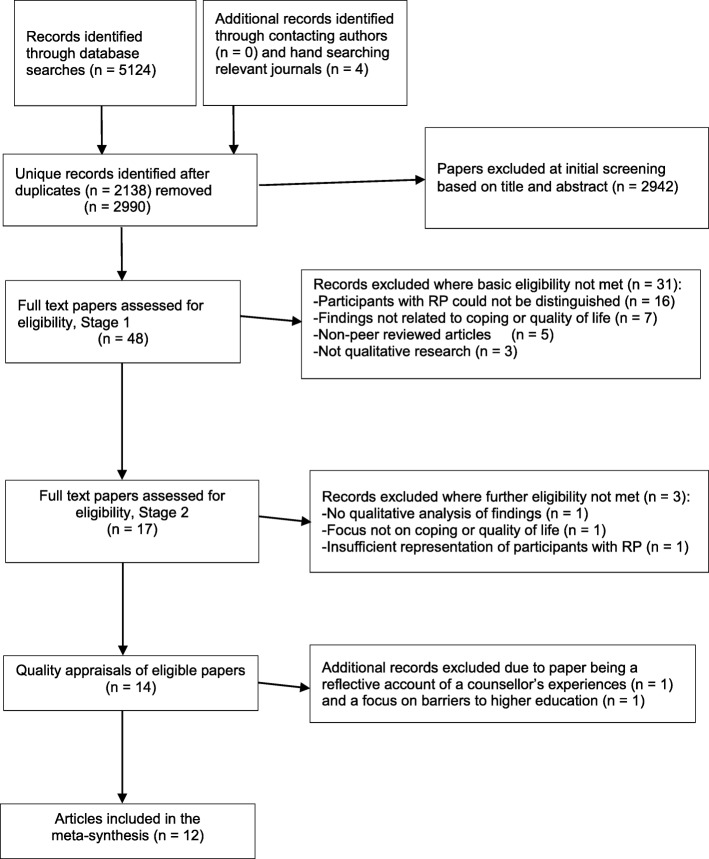
PRISMA flow chart showing literature search, study selection, and included articles for meta-synthesis

A search strategy was developed using the *Context, How, Issues of Interest, Population* (CHIP [19],) tool. The search terms or variations of the following terms were used in Web of Science, PsycINFO, PsychArticles, Library Plus, Google Scholar, MEDLINE, CINAHL, PubMed, and the Cochrane Library databases: ‘retinitis pigmentosa,’ ‘quality of life,’ and ‘qualitative’ were used either across the full articles or as a topic depending on the search options in the databases. Table 1 presents the search terms used. Figure 1 shows the PRISMA flow chart of the identification, screening, and selection processes of articles for this systematic review and meta-synthesis.

**Table 1:** Search criteria and search terms using the CHIP tool [19]

Context	How	Issues of Interest	Population
Experiences of adults living with retinitis pigmentosa	Qualitative studies; mixed studies with a qualitative approach	Coping strategies; quality of life	Adults (aged 18 years or more) with retinitis pigmentosa
‘experienc*’ AND ‘retinitis pigmentosa*’ OR ‘retinal’ OR ‘pigmentary retinopathy’ OR ‘rod-cone dystrophy’ OR ‘RP’ OR ‘tapetoretinal degeneration’	‘qualitative’ OR ‘qualitative methods’ OR ‘interview*’ OR ‘focus group*’ OR ‘case stud*’ OR ‘ethnograph*’ OR ‘narrativ*’	‘quality of life’ OR ‘qol’ OR ‘life satisfaction’ OR ‘wellbeing’ OR ‘feeling’ OR ‘coping’ OR ‘perceptions’	‘adult’ NOT child*

*Note*. Various combinations of these search terms were used for MEDLINE, EMBASE, PsycINFO, PsycARTICLES, CINAHL, Scopus, PubMed, The Cochrane Library, Google Scholar, and Web of Science databases. Search terms were entered as ‘topic’ or ‘all text’

The search strategy was developed to identify peer-reviewed publications that met the following eligibility criteria: i) qualitative studies with adults (aged 18 and over) living with RP and, ii) the study aims focused on participants’ experiences of living or coping with RP, or RP impact on quality of life. The voices of RP participants needed to be clearly discerned in mixed samples or where RP voices were undiscernible in mixed visual impairment samples, at least 75% of participants needed to have RP to ensure that themes were mostly based on the views of people with RP. Exclusion criteria included not focusing on quality of life and coping strategies in people with RP.

### Critical appraisal

Methodological quality of eligible papers were assessed independently by the authors using the Critical Appraisal Skills Programme (CASP [16],) tool, which are designed to encourage critical assessment whilst remaining methodologically neutral. Table 2 presents the quality assessment and overall quality rating scores based on the criteria. No papers were excluded on the basis of critical appraisal; appraisal was completed not as a basis for exclusion, but to highlight potential biases or limitations with individual papers and the sample overall. Synthesising findings from a variety of studies may allow for the limitations in some studies to be offset by the strengths in other papers.

**Table 2:** Assessment of article quality based on Critical Skills Appraisal Programme tool for assessing qualitative research

Study	Is the recruitment strategy appropriate?	Researcher-participant relationship considered?	Ethical issues considered?	Rigorous data analysis?	Overall score (out of 10)
1. An et al., 2014 [20]	Yes	No	Yes	No	8
2. Bittner et al., 2010 [12]	Can’t tell	Yes	Yes	Can’t tell	8
3. Falchetti et al., 2016 [21]	Yes	No	Can’t tell	Yes	8
4. Fourie, 2007 [22]	Yes	No	No	Yes	8
5. Hayeems et al., 2005 [23]	Yes	No	Yes	Yes	9
6. Schakel et al., 2017 [24]	Yes	No	Yes	Yes	9
7. Senthil et al., 2017a [25]	Yes	Can’t tell	Yes	Yes	9
8. Senthil et al., 2017b [7]	Yes	No	Yes	Yes	9
9. Spiegel et al., 2016 [26]	Yes	Yes	Yes	Yes	10
10. Thetford et al., 2011 [27]	Yes	No	Yes	Yes	9
11. Thurston, 2010 [28]	Yes	Yes	Yes	Yes	10
12. Thurston et al., 2010 [29]	Yes	Yes	Yes	Yes	10

*Note.* Columns have been omitted where all articles were rated ‘yes’. The following questions have been omitted: “Are the research questions clear?”; “Is a qualitative methodology appropriate?”; “Is the research design appropriate?”; “Has data been collected in a way that addresses the research issue?”; “Clear statement of findings?”; and “How valuable is the research?”

### Data synthesis

Meta-synthesis is a relatively recent method for systematically examining qualitative research studies on a similar topic [30]. Meta-ethnography is a specific type of meta-synthesis and primarily aims to combine, merge, and interpret data reported in the corpus of studies [30]. The processes of meta-ethnography to conduct a meta-synthesis provides an appropriate approach for building, developing, and refining a conceptual framework on quality of life issues for people with RP, by producing higher-level abstractions and understandings, whilst retaining the richness and uniqueness of the original studies by using published verbatim quotes to guide analysis [17, 18].

The authors engaged in an inductive, iterative process of review and discussion to reciprocally extract and synthesise first and second order themes. Articles were read, re-read and the details of each of the studies were recorded. Data extraction forms were used to record details of findings coded as first and second order constructs. First order themes represent the original study participants’ interpretations of their experience (i.e. the direct quotes from participants). Second order themes are the selected study authors’ interpretations of the participants’ accounts, that is to say, themes presented in the eligible papers. All eligible papers contained first and second order themes. First and second order themes were extracted from all eligible papers included in the review, presented in a matrix, and were then organized into higher level themes. Synthesis was a cyclical process so that where new themes were identified, each paper was searched in turn for occurrence of the theme. This stage involved translating findings from the individual studies in the review to derive third-order themes, i.e. themes established as a result of the meta-synthesis (i.e., meta-themes). Lastly, we developed a new conceptual model based on our line of argument that seeks to synthesise and link the third-order constructs.

### Rigor

Both authors independently screened full text papers for eligibility with over 80% agreement based on the initial reading of full papers. The remaining papers were ambiguous with queries such as whether it was possible to discern the voice of RP participants in a mixed sample. Each ambiguous paper was reviewed and eligibility was agreed through discussion, i.e., at least 75% of the sample had diagnosis of RP. The methodological quality of included studies was independently appraised using the CASP criteria [16] with almost 100% agreement between both reviewers. Differences were resolved through discussion resulting in agreement of the methodological quality of eligible papers and overall quality rating scores of each study. To minimise potential bias when developing the conceptual framework, third-order constructs were grounded in first order constructs (participant quotes) and second order constructs (selected paper authors’ themes) using an iterative process. Both review authors independently developed third-order constructs, which revealed similar meta-themes that were merged through discussion. The resulting conceptual framework was reviewed against the first and second order constructs of the selected papers to ensure the conceptual framework captured coping strategies and quality of life issues for people with RP in each study. Findings were shared with paper authors to comment on the themes developed. The results, including the meta-themes and conceptual model, were emailed to corresponding authors of four papers published in 2016 and 2017, which were included in the current review. One author responded indicating citations to the original work were appropriate.

## Results

The aims of this systematic review and meta-synthesis were to identify coping strategies pertaining to adults living with RP and how these may be linked to quality of life. Five meta-themes were identified using an inductive approach guided by the principles of meta-ethnography to synthesise findings from twelve papers identified from the systematic search: 1) managing identity: making sense of RP and managing autonomy and independence; 2) living with RP: practical and emotional issues; 3) experiences with healthcare professionals and other social support; 4) adaptive and maladaptive coping strategies; and 5) impact of RP on work and career. In the interest of preserving paper authors’ interpretations of their findings from the eligible papers, we present data-driven meta-themes before the conceptual model of how the five meta-themes may be linked to quality of life. Two out of twelve studies explicitly aimed to explore quality of life issues in people with RP [7, 25*].

The twelve eligible studies were conducted in Australia (*n* = 2) [7, 25*], Brazil (*n* = 1) [21*], Ireland (*n* = 1) [22*], the Netherlands (*n* = 1) [24*], the Republic of Korea (*n* = 1) [20*], United Kingdom (*n* = 3) [27–29*], and USA (*n* = 3) [12, 23, 26*]. Demographic information presented here is based on participants with RP. Meta-themes were based on findings obtained from a total of 126 participants across the twelve papers. Participants’ ages ranged from 18 to 85 years. There were at least 65 females from eleven papers, as it was not possible to discern demographic information for participants interviewed from one mixed methods study [20*]. An overview of papers’ setting, aims, methods, and data analysis are presented in Table 3.

**Table 3:** Characteristics of papers eligible for inclusion in the systematic review and meta-synthesis

Record number	Authors, Year	Country & setting	Research aims	Sample: *N,* characteristics*,* gender, age, ethnicity	Data collection method, average interview duration (mins), interviewer	Data analysis
1	An et al., 2014 [20]	Republic of Korea, Korean RP Society	To investigate health behaviours of people with RP, qualitatively exploring reasons for unhealthy diet and physical inactivity	*N* = 374 (187 RP; 187 matched control), *n* = 5 RP interviewed, 247 males, *x*_age_ = 40, Korean	Semi-structured interviews with 5 participants with RP, 120 mins, ND	Possibly inductive thematic analysis; ND
2	Bittner et al., 2010 [12]	USA, Johns Hopkins Wilmer Eye Institute	Understand how people with RP cope with and manage the stress generated from progressive vision loss and fluctuations in vision in daily life and explore preferences for different coping strategies	*N* = 8 legally blind adult RP (out of 10 invited); 6 Caucasian females, 2 African-American males; x = 49 (27–63) years old	Online focus groups (2, 3, 3 participants), 90 mins, 2 moderators with one experienced, but no previous experience with RP patients	Possibly inductive thematic analysis; ND
3	Falchetti et al., 2016 [21]	Brazil, Dorina Nowill Foundation	To examine the vulnerability perceived by blind consumers in the marketplace	*N* = 16 (4 females), age range from 15 to 67; *n* = 1 RP, female, age 50, social worker, married	Semi-structured interview, 55 mins, ND	Interpretative phenomenological analysis (Smith and Osborn, 2007)
4	Fourie, 2007 [22]	Ireland	Self-study to better understand researcher’s own personal identity and attitudes to blindness following a diagnosis of RP, to transform private experience in to public insight and solutions	*n* = 1, male, 36 at time of diagnosis, academic	Analysis of 12 emails to family, friends and colleagues pertaining to RP and issues around blindness experiences from 3 months prior to 3 months after receiving a long white mobility cane, researcher as participant	Self-study according to guidance by Bullough and Pinnegar (2012)
5	Hayeems et al., 2005 [23]	USA, Baltimore chapter of the Foundation Fighting Blindness and the Wilmer Eye Institute at Johns Hopkins Hospital	To explore the process of adjusting to RP and develop a model for ophthalmologists to consider and lessen patients’ suffering.	*n* = 43 adults (out of 88 invited); 24 male: 19 female; *x*_age_ = 48 (SD = 21,79); 93% White, 7% African American	Semi-structured interviews (45 mins), focus groups (2 h), 2 trained moderators	Interview and focus group data combined, and analyzed according to Straus and Corbin (1998)
6	Schakel et al., 2017 [24]	The Netherlands, two large Dutch low vision rehabilitation organizations	To explore the patient perspective of perceived symptoms, causes, consequences, and coping strategies to deal with fatigue in Dutch adults with visual impairment.	*n* = 16 (out of an initially 21 interested), *x*_age_ = 51 (+ or - 13); 9 male, 7 female, *n* = 4 with RP	Semi-structured in-depth interviews, 90 mins, interviewer was a male psychologist	Approach similar to framework method, combining deductive and inductive forms of analysis and a thematic analysis of data
7	Senthil et al., 2017a [25]	Australia, Royal Society for the Blind & Retina Australia	To explore quality of life issues of people with vitreoretinal diseases to develop group-specific item banks.	79 (32 hereditary retinal diseases of which 23 had RP, and 47 acquired retinal diseases), median age 57 (ranging from 44 to 69) for hereditary group, *n* = 23 with RP	Semi-structured interviews (45 mins), female lead author as interviewer	Phenomenological approach to data analysis
8	Senthil et al., 2017b [7]	Australia, Royal Society for the Blind & Retina Australia	Explore and understand the issues and impact of people with RP on quality of life	*n* = 23; *x*_age_ = 56 (range 28–81), 14 females	Semi-structured telephone interviews, female lead author as interviewer	Inductive analysis approach according to Straus and Corbin (1998)
9	Spiegel et al., 2016 [26]	USA	Describe the interplay between the work trajectories and the passing patterns of individuals with degenerative eye conditions in different phases of career and disease progression. Secondly, to describe the career and wellbeing outcomes associated with different work trajectories and with various concealment and passing pathways	*n* = 36 (28 with RP, 13 female); age range 42–82, *n* = 28 with RP	Face to face and telephone semi-structured interviews, interviewer was a female sociologist	Grounded theory approach to data analysis
10	Thetford et al., 2011 [27]	United Kingdom, voluntary organizations	To examine the long-term needs of people with sight loss and barriers faced by people living with sight loss in accessing the support they need over time.	*N* = 36 males, *n* = 6 with RP, with at least 1 female in her 40s based on reported case study	Face to face individual BNI and semi-structured interviews, 45 mins, ND	Biographical-narrative interpretative method (Wengraf, 2001; 2005) analyzed thematically (Gifford, 1998; Miles and Huberman, 1994)
11	Thurston, 2010 [28]	United Kingdom, Blind and Partially Sighted Society and British Retinitis Pigmentosa Society	To examine counselling experiences of blind and partially sighted adults in four core areas (mood, self-concept, social connectedness, and loss) to identify their needs, enabling recommendations for future policy and practice.	*N* = 18, ages ranged from 53 to 85, *n* = 7 with RP (4 females)	Face to face and telephone interviews lasting between 1 h to 90 min, female researcher who became legally blind later in life interviewed participants	Data analyzed using a grounded theory approach informed by Strauss and Corbin (1990)
12	Thurston et al., 2010 [29]	United Kingdom, Blind and Partially Sighted Society and British Retinitis Pigmentosa Society	To explore the emotional impact of sight loss in mood, self-concept, social connectedness, and loss	*N* = 18, ages ranged from 53 to 85, *n* = 7 with RP (4 females)	Face to face and telephone interviews lasting between 1 h to 90 min, female researcher who was legally blind later in life interviewed participants	Data analyzed using a grounded theory approach informed by Strauss and Corbin (1990)

Note: *ND* No data, *RP* retinitis pigmentosa, *N* total sample size of study, *n* sample size of participants with RP, *x*_*age*_ mean age, *SD* standard deviation

Overall quality ratings using the CASP tool of the articles were high, ranging from 8 to 10, with 10 being the maximum rating. Three of the twelve papers did not explicitly state how data were analysed, and seven studies did not report considerations relating to the researcher-participant relationship. The next section presents an overview of each of the five meta-themes identified followed by a conceptual model of how the meta-themes link with quality of life.

### Managing identity: making sense of RP and managing autonomy and independence

This meta-theme relates to paper themes on people with RP managing their identity from first noticing symptoms [28*] to a diagnosis of RP, the adjustments and re-adjustments needed to make sense of RP, and to manage autonomy and independence [7, 12, 20, 22, 23, 26, 28, 29*]. Upon diagnosis, common experiences included shock, fear, panic, disbelief, devastation, negative emotional states, and a loss of confidence related to progressive loss of vision signifying loss of independence and freedom, as well as loss of future plans for some [12, 22, 28, 29*]. Paper themes reported that people with RP search for meaning to understand why RP has happened to them, including searching for genetic or non-genetic explanations, or turning to spiritual meaning-making [23, 28*].

A diagnosis of RP meant that a number of activities and behaviours an individual would engage with may no longer be possible which could have implications for their sense of identity [12, 28, 29*]. Feelings of loss were likened to experiencing bereavement and included, reports of loss of vision, hobbies and pastimes (e.g. reading), loss of social support leading to feeling isolated or not being understood by others [22, 28, 29*]. People with RP also reported tension in their fight to maintain independence as vision worsened, and some felt like a burden or nuisance to others for completing daily tasks [7, 12, 22, 26, 28, 29*]; for some, relying on others was perceived as a major inconvenience [7*]. To counteract these feelings, some people used assistive technology and devices to maintain independence [12, 22*].

Some people with RP had reservations around using a mobility cane, which was seen as symbolic of loss of autonomy. Despite reluctance to use a mobility cane by some people with RP [22, 28*], those who used one reported improvements in their mobility and felt this allowed for greater exploration of one’s environment [22*]. Some experienced needing to act blind enough to warrant use of a mobility cane [22*].

People with RP resolve their personal identity either by self-identifying as sighted and engage in hiding and passing behaviours to cover-up visual impairment if they could get away with it, or they were accepting of the RP condition and were open about the condition with others [22, 23, 26*]. Some people with RP refer to a transition from being sighted to being a person with visual impairment, and that self-esteem was an important enabling construct of this transition to a person accepting their visual impairment [23*].

This meta-theme highlighted the emotional and psychosocial experiences linked to making sense of RP, adapting to a changing sense of identity, and negotiating the need for independence and reliance on others or tools (e.g. mobility cane). These intra-individual factors are closely linked with another intra-individual meta-theme related to living with RP.

### Living with RP: practical and emotional issues

This meta-theme relates to the lived experiences of people with RP and identifies a number of factors people need to cope with [7, 12, 20, 22, 24–29*]. People with RP experience difficulty performing day-to-day tasks such as reading, seeing in changing light conditions, shopping, driving, playing sports, taking part in leisure activities, and doing household chores [7, 12, 20, 22, 25*]. These issues are interrelated and have an effect on lifestyle choices; for example, where people experienced difficulty shopping for food, they opted to eat fast food options [20*]. People who feared bumping into others in exercising facilities cited this as a reason for being physically inactive [20*]. For practical reasons, some people with RP chose to continue driving well beyond when they should have stopped driving and only gave up after having an accident or a very close call [12*]. The external environment and limited transport options made navigating, traveling or going out alone difficult for some people with RP, resulting in people having more complex travel plans [7, 12, 28*].

In addition to practical challenges, living with RP is also accompanied by fatigue, and emotional and psychological states, such as fear, isolation, and vulnerability in relation to the aforementioned practical issues [7, 12, 20, 24, 25, 27, 28]. Fatigue may also be involved in diminishing emotion regulation capacity, adding to the psychological strain of living with RP [6]. People with RP not only deal with their own judgements but also with stigma they perceived others had about them due to their visual impairment [20, 22, 26, 28, 29]. Some participants reported being patronized or treated by others as if they had low intelligence [28]. Social interactions are reported as challenging due to, in some cases, inability to identify social cues, which could result in less participation in social events [7].

This meta-theme presented a number of practical and emotional issues people with RP need to cope with, as well as illustrating a ripple effect of how visual difficulties may lead to unhealthy lifestyle choices, which can be detrimental to people’s health. The next meta-theme focuses on an inter-individual theme, which is likely to influence and be influenced by intra-individual meta-themes.

### Experiences with healthcare professionals and other social support

Papers reported experiences of people with RP with various healthcare professionals, assumptions about formal support services, and informal interactions with others (e.g., family, friends, supermarket attendants, etc.). In terms of the diagnostic experience, physicians and clinicians’ language use with newly diagnosed people with RP could broadly be categorised into three groups; blunt, vague, and mild, was reported to lead to feelings of devastation, anxiety, and hopefulness respectively [23]. Participants often turned to healthcare professionals or others with RP to help make sense of the diagnosis at an intellectual (e.g. factual information), practical (e.g. how to prepare to live with RP) and emotional (e.g. how to deal with the emotional aspects of the condition) level [28, 29], though the need and importance of each of these levels of information varied from participant to participant [28].

Despite resistance or negative perceptions related to counselling, psychologists, and therapy [29] by those who had not engaged with these services, those people who had participated in counselling reported finding it helpful to alleviate anxiety [22] and to adopt a more helpful perspective around RP [29]. In fact, despite the resistance to counselling, participants would have liked the opportunity to talk about their feelings at the time of diagnosis [29]. Some people with RP required clarification around the purpose and expected outcomes of counselling [28]. The power imbalance between patients with RP and clinicians lead to feelings of lack of autonomy [28]. For some participants it was important for the counsellor to also be living with a visual impairment to ensure participants felt they were truly understood by the counsellor; distrust of sighted counsellors were mentioned [28]. One author reported that people with RP may be quicker to open up to a person with a similar progressive visual condition compared to others without the condition [26].

Support groups of people with RP could be helpful, however in one study a female participant in her 40s reported that a support group of mostly older people did not meet her needs [27]. This case study indicates reliance on family members for daily activities may result in some people with RP not taking up opportunities for rehabilitation or training to develop the skills to enable unassisted living [27]. Not feeling understood by healthcare professionals or members in support groups could lead to feelings of isolation [28, 29]. People with RP wanted to have good role models that they could take as an example for living with RP [28, 29]. People with RP also reported the need to understand their legal position in society, statutory protection, and constitutional rights [22].

This meta-theme presented various interactions and encounters with healthcare professionals and services, as well as social support groups and informal others, that could be helpful or unhelpful to people living with RP. The next meta-theme presents coping strategies adopted by people with RP to deal with the challenges of RP and the experiences of living with RP in relation to the preceding three meta-themes.

### Adaptive and maladaptive coping strategies

Living with RP has its challenges as identified in the preceding three themes. Papers reported people living with RP adopt various adaptive and maladaptive coping strategies, which are presented in this meta-theme. Adaptive coping strategies included examples of problem-focused coping strategies, where actions were taken to make a situation achievable. Emotion-focused coping strategies were related to accepting that behaviourally there is nothing an individual can do to change the situation and therefore people chose to deal with their emotions.

Problem-focused coping strategies to deal with challenges faced as a result of vision impairment included, scheduling events to coincide with when they had best vision and to plan ahead [12, 29], allowing more time when travelling to new and unfamiliar places [7], using gadgets and tools for reading, shopping, and moving around were helpful [7], and being slower and more careful when carrying out daily tasks [7]. In anticipation of potential challenges one participant reported getting a haircut, demonstrating an active approach to dealing with progressive vision loss [28]. Communicating with others who have RP was reported as being helpful [12, 22], as was obtaining factual information about RP from the experiences of others with RP to know what to expect [22]. Communication is key for people with RP to explain to family, friends, colleagues, and others what practical and emotional support they may need for their visual impairment [12, 21, 22, 27], though this may be difficult in ensuring those without progressive vision impairment understand what it is like to live with RP [7, 25]. For example, due to the nature of RP, it may be possible for some people to be able to read but require a cane for mobility, which may need to be explained to others [22]. These conversations could help resolve the conflict between social stigma around what people with RP can or cannot do, and allow people with RP to use canes that can enable mobility [22]. Some people with RP stated that relying on social support from early on in their RP diagnosis made it easier to adapt to vision loss and deal with social encounters [21].

In terms of emotion-focused coping strategies participants reported engaging in downward social comparisons to keep their vision loss in perspective by comparing this to worse conditions [12], this helped participants appreciate what they had and the abilities they retained [12]. Humour and laughter, in particular, the ability to laugh at self was reported as helping deal with RP [12]. In participants who experienced severe fatigue, the cons of feeling tired were outweighed by the benefits and positive feelings related with socialising [24]. Being socially integrated was reported by people with RP as being therapeutic [28, 29]. Some participants were hopeful of a cure for RP being discovered in their lifetime [12]. Some participants felt it was important to give themselves permission to vent, gripe and complain to relieve stress and cope with RP [12]. People dealt with the transition from being sighted to being a person with visual impairment differently [28, 29]. Where participants had experience in both engaging in passing behaviours for a person with sight, upon accepting visual impairment and being open about this, people reported this being less distressing [26]. Some participants found solace in religious faith [28, 29].

Some participants engaged in maladaptive coping strategies, which could include avoiding addressing a problem or accepting one’s condition, or engaging in behaviours that are harmful to health to deal with living with RP and could perhaps exacerbate the challenges of living with RP. For example, one study reported a participant taking up smoking [29], another reported people with RP eating more fast food and engage in less physical activity during the transition from sighted to a person with a visual impairment [20].

This meta-theme presented examples of mostly adaptive (behavioural, e.g. giving oneself more time when traveling and going to new places, and psychological, e.g. acceptance of RP, and communicating one’s abilities and where people with RP need support with others) and some maladaptive coping strategies identified among people with RP. Decisions to adopt a specific coping strategy are likely to be influenced by intra- and inter-individual factors. As such, this meta-theme is presented as a construct in its own right, to represent that chosen coping strategies can influence intra- and inter-individual factors and vice versa. The next meta-theme specifically focuses on the impact of RP on work and career context.

### Impact of RP on work and career

For people with RP, the impact of RP on work and career trajectories is noteworthy, as participants are typically diagnosed when they are relatively younger compared to later-onset visual impairments (e.g. age-related macular degeneration). The issues noted in this meta-theme are related to preceding themes presented, however it is worth clustering work and career related issues in this way for a more comprehensive picture of the impact of RP on interactions with others and organizational systems linked to a work and career context, and the influence this may have on coping strategies and quality of life.

It has been reported that people with RP frequently need to change jobs to suit their abilities as their eye condition worsens [7], and for some, finding work was a struggle amidst reduced job opportunities [7, 26]. For some people with RP, difficulty with employment led to financial constraints [7]. Papers reported people with RP have a sense of worry over their careers and how useful they were or could be in the workplace, and whether workplaces would accommodate their visual impairment [7, 26]. Some people with RP did not believe workplaces would make the necessary adaptations to accommodate an individual with progressive visual impairment [22, 26]. Some people with RP chose to conceal RP and engage in normalising behaviours for fear of discrimination, termination, or being treated as less capable or incompetent in the workplace [26].

There were assumptions that career paths chosen prior to the RP diagnosis would not be possible as vision declined and therefore adjustments were made to career plans that would be more accommodating of one’s RP condition [26]. For some, no adjustments were made to one’s career trajectory and they opted to conceal their RP status in the workplace [26]. Even after revealing their condition in the workplace, some people remain conflicted about their identity between being considered ‘normal’ versus a person with disability but generally there was a sense of relief and stress reduction after revealing RP status [26]. One of the main reasons for concealing RP status was related to the lack of protective laws and workplace culture [26]. Some participants were always open about their RP status in the workplace, even when they could have engaged in passing or normalising behaviours [26]. Some people with RP were only comfortable revealing RP once a secure and relatively high status had been reached in the workplace [26]. For some people with RP, taking early retirement was a solution to career related issues [26].

This meta-theme can be grouped with inter-individual factors and presents the concerns around continuing with career plans prior to the RP diagnosis, finding a suitable employment position, and the willingness of workplaces to adapt to the needs of a person with RP.

#### Conceptual model of factors influencing coping and quality of life in people with RP

**Fig. 2:**
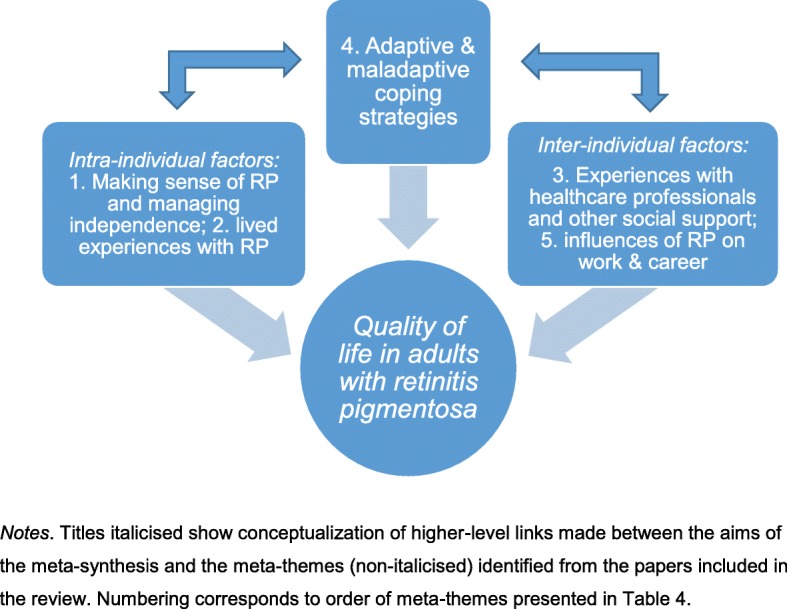
Conceptual model of how meta-themes may relate to influence coping strategies and quality of life in people with RP. *Notes*. Titles italicised show conceptualization of higher-level links made between the aims of the meta-synthesis and the meta-themes (non-italicised) identified from the papers included in the review. Numbering corresponds to order of meta-themes presented in Table 4

Five meta-themes were identified that can be clustered as intra- and inter-individual factors. Psychological and emotional factors related to living with and making sense of RP, and the experiences related to maintaining and negotiating independence comprised intra-individual factors. Meta-themes on the experiences of people with RP with healthcare professionals, legal issues, other social support, and influences of RP on work and career trajectories made up inter-individual factors. Together these intra- and inter-individual factors were identified as likely influencers of people’s decisions of coping with RP. Likewise, chosen coping strategies of people with RP are likely to influence people’s experiences related to intra- and inter-individual factors. These meta-themes are interrelated and complex, which has implications for how best to explore their impact on quality of life. People’s perceptions of their quality of life may in turn influence their lived experiences with RP and the interactions and opportunities they encounter with others in formal and informal social functions and settings. A summary of the meta-themes are presented in Table 4 and the conceptual model is presented in Fig. 2.

**Table 4:** Themes from articles informing the development and definitions of meta-themes

Meta-synthesis theme titles and definitions	Themes from eligible papers	Articles^a^
*1. Managing identity: Making sense of RP and managing autonomy and independence* Emotional and psychosocial intra-individual experiences linked to making sense of RP, adapting to changing sense of identity, and negotiating the need for independence and reliance on others or tools.	Responses to diagnosis shock, fear, panic, disbelief, devastation, negative emotional states, and loss of confidence, loss of independence and freedom, loss of future plans, searching for reasons why RP has happened to them, acceptance of activities that may no longer be possible due to vision impairment, likening sight loss to bereavement, tension in fight to maintain independence as vision worsens, feeling like a burden to others, reliance on others perceived as an inconvenience, using assistive technology to maintain independence, mobility cane symbolic of loss of independence and also enables greater exploration of one’s environment, acting blind enough to warrant cane use, the transition from being sighted to a person with vision impairment, acceptance of vision impairment.	1, 2, 4, 5, 8, 9, 11, 12
*2. Living with RP: Practical and emotional issues* Practical and emotional issues people with RP need to cope with on a daily basis at an intra-individual level.	Difficulty performing daily tasks, e.g. reading, seeing in changing light conditions, shopping, driving, playing sports, taking part in leisure activities, doing household chores, preparing healthy meals, need for more complex travel plans due to challenges in the external environment, fatigue, fear, isolation, vulnerability, diminished emotion regulation capacity, judgements and stigma of self and from others about people with vision impairment, being patronized or treated as if they had low intelligence from others, social interactions challenging due to inability to identify social cues resulting in less participation in social events.	1, 2, 4, 6, 7, 8, 9, 10, 11, 12
*3. Experiences with healthcare professionals and other social support* Inter-individual experiences of people with RP with healthcare professionals, assumptions about counselling and therapy, other formal support services, and interactions with informal others.	Not feeling understood by healthcare professionals or members in support group leading to isolation, language used by physicians/clinicians led to feelings of devastation, anxiety, and hopefulness, need for factual, practical, and emotional information at time of diagnosis from healthcare professionals, resistance to counselling and therapy, counselling helpful to alleviate anxiety, wanting opportunities to talk about feelings at the time of diagnosis, clarification and expected outcomes related to counseling, power imbalance between patient and clinician leading to feelings of lack of autonomy, distrust of sighted counsellor, more trusting of counsellor living with a visual impairment, reliance on family members for daily activities, need for good role models living with RP, understanding legal position in society, statutory protection, and constitutional rights.	4, 5, 9, 10, 11, 12
*4. Adaptive and maladaptive coping strategies* Adaptive (problem-focused and emotion-focused strategies) and maladaptive coping strategies reported to be used by people living with RP.	Problem-focused coping strategies: scheduling events to coincide with best vision, planning ahead, allowing more time in new and unfamiliar places, using gadgets and tools for daily activities, communicating with others who have RP, obtaining factual information about RP, communicating with others about practical and emotional support needs; emotion-focused coping strategies: downward social comparison, being grateful for abilities retained, humour and laughter, social integration, hope for a cure, permission to vent, gripe, and complain for stress relief, being open about RP less stressful than engaging in passing behaviours for a person with sight, faith; maladaptive coping strategies: smoking, eating unhealthily, and reduced physical activity.	1, 2, 3, 4, 6, 7, 8, 10, 11, 12
*5. Impact of RP on work and career* The impact of RP on interactions with others and organizational systems linked to a work and career context, and how these may influence coping strategies and quality of life.	Changing jobs; finding jobs to suit ability as eye condition worsens; reduced job opportunities; challenges with employment led to financial constraints; sense of worry over career; concerns over being useful in the workplace; concerns over workplace being accommodating to progressive vision loss; concealing RP for fear of discrimination, termination, or being treated as less capable or incompetent; relief and reduction in stress after revealing RP status to others; lack of protective laws as reason for not disclosing RP status; revealing RP status once in a secure and relatively high status; taking early retirement.	4, 8, 9

*Note*. ^a^ Article numbers correspond to numbering presented in Tables 2 and 3

## Discussion

The aims of this systematic review and meta-synthesis were to present an overview of adults living and coping with RP, and to present how these findings may inform the development of interventions to improve quality of life in this population. Qualitative studies exploring the lived experiences of people with RP were meta-synthesised to present issues pertinent to adults living with RP. The conceptual framework presents an interpretation of how the identified meta-themes may interact to influence quality of life. The findings are similar to psychosocial factors associated with adaptation to chronic illness and disability [31] but are derived specifically from the unique experiences of adults living with RP. We recognise that there are different definitions of quality of life [10, 11] and coping could be a domain of quality of life itself for the RP population based on the definition of quality of life we have adopted. However, coping is a multidimensional construct; our definition of coping does not only focus on outcomes, such as participants ability to cope with the challenges of living with RP, our definition also focuses on the process of coping, such as the use of specific coping strategies that result in the quality of life domains in line with our definition. Based on the broad definition of quality of life adopted in this paper, we identified a number of intra- and inter-individual factors that are related to a person’s subjective perceptions of the impact of RP on their physical, psychological, social functioning, and well-being. The meta-themes identified in this paper related to quality of life in people with RP were: 1) varied physical and psychosocial factors associated with changes in identity related to transitioning from a person with sight, to a person with visual impairment as the condition progresses; 2) day-to-day practical and emotional experiences people need to cope with; 3) the challenges and opportunities related with healthcare professionals and services, as well as other social support (e.g. family, friends, encounters with informal others in social settings); 4) adaptive and maladaptive coping strategies used to live with RP; and 5) the influence of RP on work and career paths.

Lazarus and Folkman’s Transactional Theory of Stress and Coping [9] is the most widely used model of how people adapt to living with a chronic progressive condition. According to this model, people first make an appraisal of the stressor (i.e. primary appraisal), in this case, diagnosis and experience of living with RP, and then make a secondary appraisal of their own ability to manage the stress, before subsequently deciding on how they will cope with the stressor (i.e. adopting a coping strategy). The model is helpful for explaining how people with RP may choose their coping strategies. The model, however, does not explicitly account for intra-individual factors, such as self-esteem, mental health, and managing identity, or inter-individual factors, such as interactions with healthcare professionals, family, and friends, nor does it take into consideration the interrelationships between these factors and the mechanisms through which they may influence appraisals. This presents various opportunities for theory and intervention development to improve quality of life in this population. Another finding not supported by the model relates to developing ways to improve better social integration of people with RP, namely through work and facilitating travel, which is likely to enhance quality of life. Some papers reported participants not being physically active due to obstacles and barriers encountered in exercise facilities, suggesting a need for indoor and outdoor exercising facilities that are conducive to people with RP engaging in physical activity. Modifying the environment can offer people with RP more means of control which could minimise dependency and feelings of powerlessness [32] resulting in increased independence and quality of life.

Most coping strategies identified in this paper can be categorised as adaptive coping strategies. This could be reflective of the fact that participants who typically self-select to take part in research may be more likely to successfully self-manage RP, compared to people with RP who do not volunteer to take part in research [33]. In some papers, participants were recruited from support groups or organizations, which may better equip participants to choose adaptive coping strategies compared to those living with RP who are not in support groups.

Based on the findings of this meta-synthesis, it is possible to identify factors that are within the control of people living with RP, which has implications for developing interventions to help people with RP choose adaptive coping strategies to self-manage RP. Education can strengthen the sense of control over living with RP, reduce feelings of confusion and enhance decision making [34]. Equipping people with RP with the knowledge about the uncontrollable aspects of RP (i.e. presently incurable), and exploring the applicability of psychologically-based interventions to manage emotional experiences related to RP could be helpful. Self-management programmes based on enhancing self-efficacy are successful in promoting behaviour change in a number of chronic conditions [35]. Self-efficacy could be considered a determinant in the appraisal of RP and the coping strategy used [36]. Ensuring people with RP develop skills and self-efficacy to choose adaptive coping strategies could ensure that as the condition progresses, people are better equipped to adapt to the changes they encounter. This highlights the importance of balancing facilitative social encounters and support with some level of individual acceptance, adjustment and re-adjustment to RP for doing daily activities.

Acceptance was seen as important for coping with RP in the papers included in this review. Acceptance can be conceptualised as an adaptive emotion-focused coping strategy because it is effective at minimising stressors [9, 31]. Interventions that combine cognitive-behavioural strategies and meditation awareness training can be used to teach acceptance and coping skills to people with RP to manage the psychological impact related to the condition [37]. These interventions could include behaviour change techniques such as goal setting in line with disease progression and nature of condition, pacing activities to minimise fatigue, relaxation techniques, cognitive therapy, communication skills to manage encounters in workplace, social or healthcare settings, and the management of high-risk situations (e.g. isolating self from social encounters and others due to visual impairment). The lack of publications on the applicability of psychological interventions for people with RP suggests this is a potential avenue for intervention development and research.

Acceptance of the RP condition also has implications for re-evaluating one’s identity. Loss of self without the development of an equally valued new self can contribute to the sense of powerlessness experienced [38]. The illness experience of living with a chronic condition can result in an identity crisis which involves comparisons between former selves and ‘ill selves’ [36]. Psychosocial interventions that include reappraisal of personal identity could support people with RP to adapt and adjust to living with RP.

Next, we present a number of implications for healthcare providers working with people with RP and policy makers for workplace settings to better support people with RP to remain or become integrated into work.

### Implications for healthcare providers

As people living with RP wait for a cure, one way to improve quality of life is to ensure individuals develop the psychosocial and behavioural strategies and skills to manage the day-to-day challenges of living with RP and to establish and maintain a harmonious set of relationships [39]. The findings suggest that some people with RP are unsatisfied with the interactions with healthcare professionals, in terms of information received related to factual, practical and emotional aspects of RP, as well as how this information was conveyed (i.e. blunt, vague, mild). Some papers identified a power imbalance between patients and healthcare professionals. Training healthcare professionals to adopt a patient-centred approach to the medical consultation ensures the patient is an active participant in the process, who is listened and responded to appropriately [40], which could minimise some of the distress people with RP experience at the time of diagnosis.

Bandura’s Social Learning Theory and vicarious learning, in its simplest form, posits that individuals can learn new behaviours from others [41]. There is a lack of role models for people with RP which could influence adaptive coping behaviours, levels of functioning and individuals’ definitions of themselves to their limitations [42] due to living with RP. Social support is related to positive adaptation and is used as a coping strategy which can enhance self-efficacy and provide emotional support [35]. Healthcare professionals can also discuss the possibility of joining support groups as a possible means for adopting a role model. People with RP look to healthcare professionals, as well as others living with RP for guidance, inspiration, and support to self-manage their condition. Healthcare professionals, such as counsellors, clinical and health psychologists, are well suited to work with people with RP to accept the condition, set manageable and realistic goals with the intention of encouraging pacing and minimising fatigue and burnout in this population. Healthcare professionals and support organizations are also well suited to encourage and help maintain regular physical activity, as this behaviour was reported as having positive physical and psychological impact in people with RP. Support groups can play a crucial role in ensuring people newly diagnosed with RP are introduced to others with RP who may act as role models. Healthcare professionals can discuss joining RP support groups with individuals.

Some people with RP talked about mistrust they had for counsellors who were not living with a visual impairment, and that people with RP did not feel they could truly be understood by those not living with the condition. Educating people with RP about the purpose and format of counselling may minimise or alleviate concerns related to the perceived need for counsellors to be living with a visual impairment for counselling to be meaningful to people with RP who may benefit from these services.

Healthcare providers and support organizations that take these factors into consideration may positively influence the quality of life in people with RP. The Guided Care model, which was originally developed to improve the quality of life for older adults [43], could be adapted by healthcare providers to support people with RP by retaining the following principles of chronic care: disease management, self-management, case management, lifestyle modification, transitional care, and caregiver education and support. Adopting a multi-disciplinary, holistic approach to care would enable healthcare providers to conduct appropriate assessments, planning, self-management support, monitoring, psychosocial support, educating and supporting caregivers, and facilitating access to resources, which could improve the quality of life and coping strategies used by people with RP. This approach will ensure people with RP have the information required at the time of diagnosis and during progression of the condition when visual functioning becomes increasingly impaired to maximise functioning and independence.

### Suggestions for work and career support for people with RP

The suggestions presented in this section are intended to shape policy agendas in government departments or ministries of work in the countries included in this review, namely Australia, Brazil, Ireland, Netherlands, Republic of Korea, United Kingdom, and USA. People with RP experienced challenges around finding, maintaining, or changing work, suggesting that at the time of RP diagnosis, support with career planning may be helpful to some individuals to develop realistic expectations and goals in the workplace. Three papers identified concealing behaviours from people with RP to avoid disclosing their condition for fear of being discriminated against, considered incompetent, or risking losing one’s job [7, 22, 26]; this is a barrier to developing adaptive coping strategies. The impact RP may have on an individual’s work and career choices are multi-dimensional and a temporal process, meaning an individual may shift along a continuum across different points in time, and there is much variation between individuals’ openness regarding RP [26]. Supporting workplaces to be able to make adaptations for people with RP to be retained or reintegrated into the workforce, are required to overcome the challenges of pursuing a career for people with RP. The dominant culture emphasises qualities of physical health and standards of perfection that clash with chronic conditions [44] such as RP, which can exacerbate stigma in the workplace. There is a need for interventions at the workplace level to change people’s perceptions of the abilities of people with RP to create environments that are conducive to people with RP adopting adaptive coping strategies, and to minimise stigma that can be associated with inequitable treatment [44].

### Future research

There is value in comprehensively understanding the experiences of people with RP, given the earlier onset and unique progression of the condition compared to conditions such as diabetic retinopathy or age-related macular degeneration. Comparing the experiences of people with other visual impairments to the experiences of people with RP may reveal similarities that could benefit from the suggestions presented in this paper. Future research on longitudinal interviews with people with RP from diagnosis to advanced stages of RP to explore how transitions in identity are experienced, resolved, and to identify optimal points in the RP trajectory to provide relevant support to people (e.g. at the time of diagnosis). Intervention studies to promote self-management, acceptance and communication skills to minimise psychosocial distress associated with living with RP could be developed and evaluated. Training programmes targeting healthcare professionals could be developed to promote patient-centred consultations resulting in more adaptive coping strategies which increase the quality of life for people living with RP.

### Strengths and limitations

In this systematic review and meta-synthesis we interrogated the findings from twelve eligible papers based on a focused search strategy to establish five interrelated meta-themes. The conceptual model based on the meta-themes presents the relationship between the meta-themes and quality of life in people with RP. The findings of the papers included in the review were complementary and built on one another to develop a more comprehensive account of the experiences of people with RP than the studies in isolation; this is a strength of the paper. The authors engaged in a blind and iterative review process for developing the conceptual model. The search strategy was broad enough to capture studies and specific enough to exclude irrelevant ones. The overall sample was homogenous (nearly all RP) in terms of the condition of interest and heterogeneous in terms of demographic characteristics (different age range, gender, employment status, different countries, etc.). The inclusion of different countries highlights a global experience of similar experiences encountered by those living with RP.

In terms of the limitations, most participants in these studies were predominantly people with late stage RP meaning these findings may not be applicable to the spectrum of RP progression. Most studies selected participants from charity organizations, where people who may be experiencing greater challenges with RP may be more likely to join organizations to seek more information and support. Alternatively, it may be possible that people who are more accepting of their condition may be more likely to engage with such organisations. It would be worth conducting a study to explore whether there are differences in people with RP who (do not) engage with support organizations to ensure more appropriate support can be offered to those who may not choose to engage with such organizations. Some studies reported commencement of thematic data analysis until all data had been gathered, meaning identification of data saturation would not have been possible during data collection (e.g. [25]). It is recommended for thematically analysed data, an iterative process is adopted, with data analysis occurring alongside data collection to shape and revise the research question, interview guide, and to establish when saturation has been reached [45].

## Conclusions

This systematic review and meta-synthesis presents a comprehensive overview of the physical and psychosocial factors experienced by people with RP that can inform health professionals, employers, people with RP, and their family and friends. This paper offers one interpretation of a synthesis of perspectives of people’s experiences with RP, by ophthalmologists, counsellors, other healthcare professionals, and researchers working in this field. It is intended that the findings will contribute to informing the development of interventions to support quality of life in people with RP in their day to day lives and in the workplace, which can reduce financial strain and increase social functioning. This paper highlights the importance of individual preferences and the impact of psychosocial factors related to people’s experiences of living with RP, and the need for these factors to be accounted for in interventions that aim to enhance quality of life in this population.

### Summary

What was known before?

- Retinitis pigmentosa (RP) is an incurable group of inherited eye conditions, affe
- cting 1 in 4000–5000 people and is the leading cause of inherited blindness in people under the age of 60.
- People with RP become more dependent on others for daily tasks, are less likely to contribute to the workforce and society, and are more likely to use health services compared to those without the condition.
- People with RP experience physical (e.g. loss of visual function), social (e.g. limited or no employment opportunities), psychological (e.g. depression), and emotional (e.g. devastation and vulnerability) challenges which influence their quality of life, more so than people with other visual impairments such as age-related macular degeneration.

What does the review add?

- Isolated studies of people’s experiences with RP have been meta-synthesised to present a more comprehensive narrative of the complexities of the multidimensional intra- and inter-individual factors influencing choice of coping strategies and quality of life.
- Policies are needed to ensure the workplace and public settings are conducive for people with RP to engage in daily activities.
- For people living with RP, behavioural and psychoeducational interventions focusing on acceptance of the condition, communication skills to explain abilities and help needed from others, planning ahead for dealing with progressive vision impairment can be offered to develop skills to self-manage RP.
- For healthcare professionals, interventions for raising awareness of the impact of communication style on people’s emotional states and variations in people’s need for factual, emotional, practical information related to the RP diagnosis could be developed to help facilitate better adaptation and coping with the condition.

## Data Availability

The datasets used and/or analysed during the current study are available from the corresponding author on reasonable request. Supplementary materials have been provided to facilitate interpretations, replications and for building on findings reported in the article. The raw data used in this systematic review were the primary research papers that have been cited in the reference list.

## Abbreviations

CASP: Critical Appraisal Skills Programme
CHIP: Context, How, Issues of Interest, Population
PRISMA: Preferred Reporting Items for Systematic Reviews and Meta-Analyses standards
PROSPERO: Predetermined registered protocol
RP: Retinitis pigmentosa
